# Supplementary material for: Extreme rainstorms drive exceptional organic carbon export from forested humid-tropical rivers in Puerto Rico
Source: Nat Commun. 2022 Apr 19;13:2058. doi: 10.1038/s41467-022-29618-5 (PMC9018737; doi:10.1038/s41467-022-29618-5)
Supplement: Supplementary file 1 — Supplementary Information [file 41467_2022_29618_MOESM1_ESM.pdf]

Supplementary information for

Extreme rainstorms drive exceptional organic carbon export from forested humid-tropical rivers in Puerto Rico

K. E. Clark, R. F. Stallard, S. F. Murphy, M. A. Scholl, G. González, A. F. Plante, and W. H. McDowell

Abstract in Spanish

Los eventos extremos de precipitación en las Montañas de Luquillo, Puerto Rico, exportan la mayor parte del sedimento suspendido y del carbono orgánico particulado. Tras el análisis de 25 años de datos de carbono del río y sedimento suspendido, que tenían como objetivo los efectos de huracanes y otras grandes tormentas, estimamos el carbono orgánico particulado biogénico en 65 ± 16 tC km^{-2} $año^{-1}$ para el río Icacos y 17.7 ± 5.1 tC km^{-2} $año^{-1}$ para el río Mameyes. Estas cuencas graníticas y volcanoclasticas funcionan como importantes sumideros de dióxido de carbono atmosférico, en mayor medida a través de la exportación de carbono orgánico particulado biogénico durante tormentas extremas. Comparado con otras regiones, estas altas cantidades de carbono orgánico particulado biogénico están acompañadas por cantidades de sedimentos suspendidos menores. De esta forma, la exportación de carbono orgánico particulado de estas cuencas ha sido infra valorado por relaciones de cantidades previas, las cuales se derivan principalmente de cuencas con rocas sedimentarias fácilmente erosionables. Es por esto que los ríos que drenan de una roca madre pobre en carbono petrogénico requieren ser contabilizados de forma separada para estimar sus contribuciones al ciclo geológico de carbono.

Figures

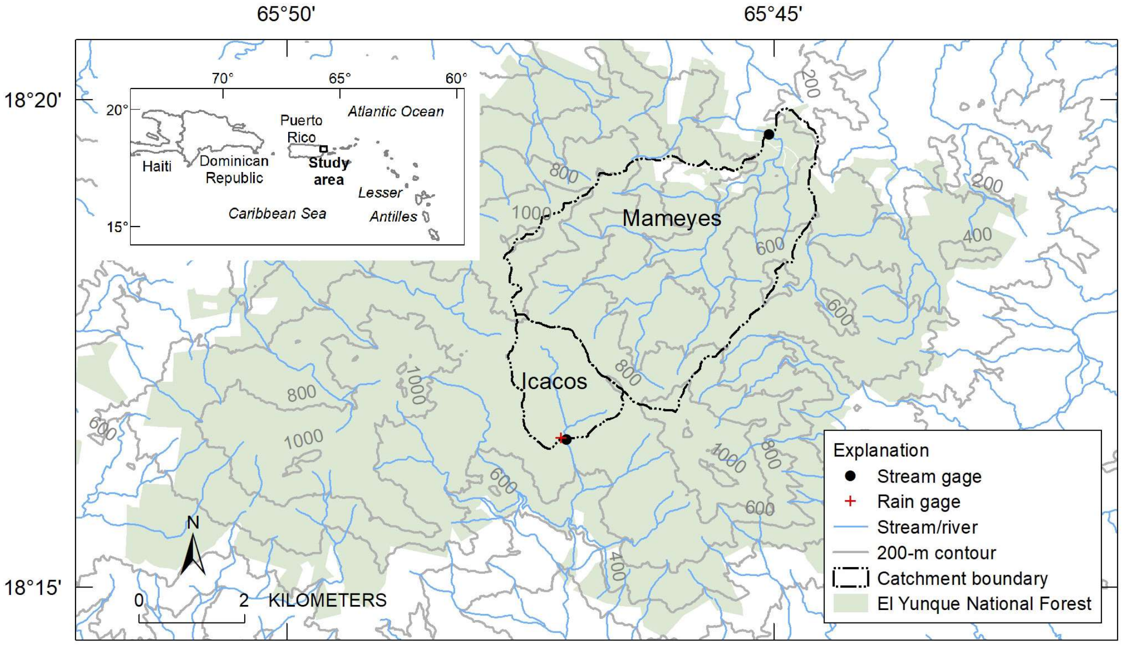

Supplementary Figure 1: Map of the study catchments, the Mameyes and Icacos. They are located in El Yunque National Forest, in Puerto Rico. The inset map shows the location of Puerto Rico, with study area indicated (black box).

a

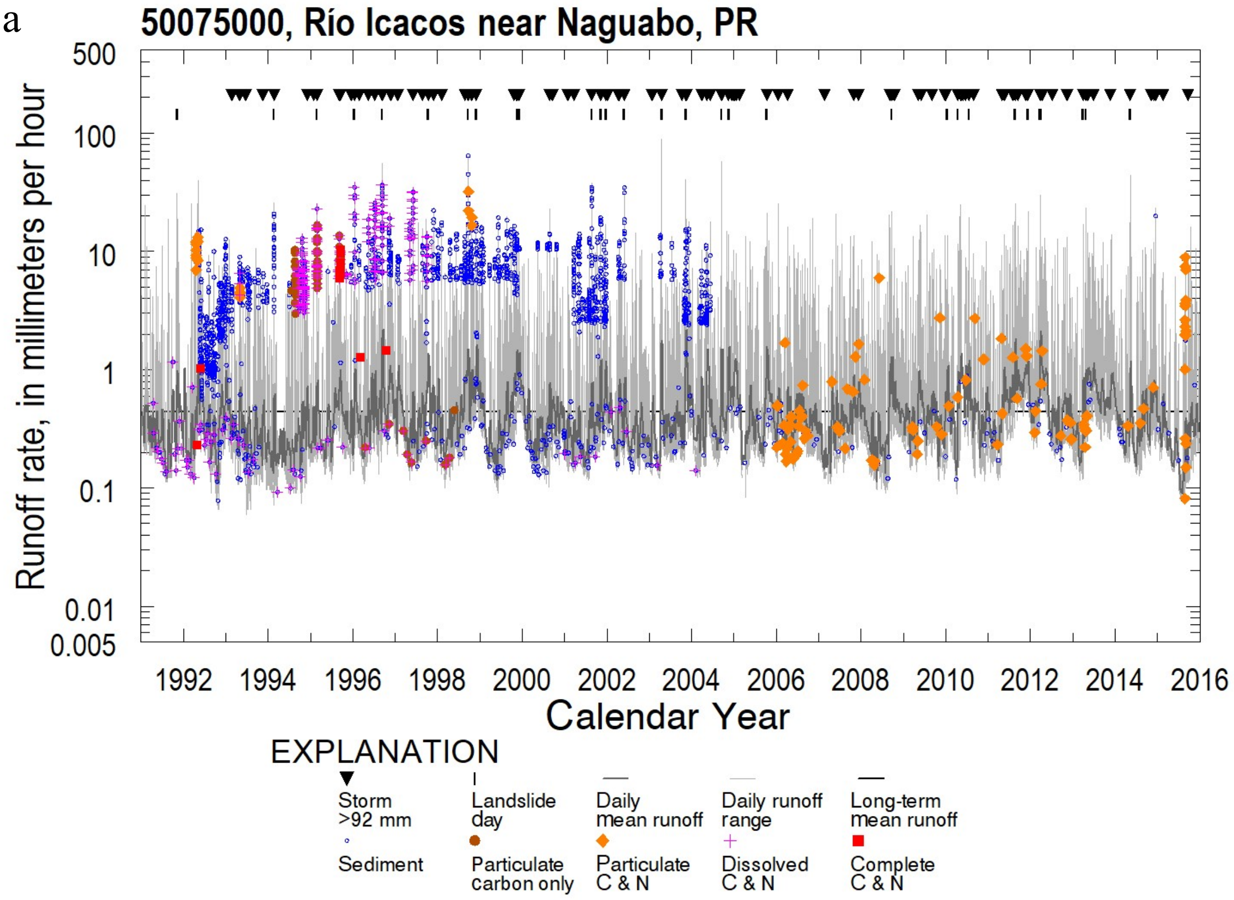
50075000, Río Icacos near Naguabo, PR
Runoff rate, in millimeters per hour
500
100
10
1
0.1
0.01
0.005
1992
1994
1996
1998
2000
2002
2004
2006
2008
2010
2012
2014
2016
Calendar Year
EXPLANATION
Storm >92 mm
Landslide day
Daily mean runoff
Daily runoff range
Long-term mean runoff
Sediment
Particulate carbon only
Particulate C & N
Dissolved C & N
Complete C & N

b

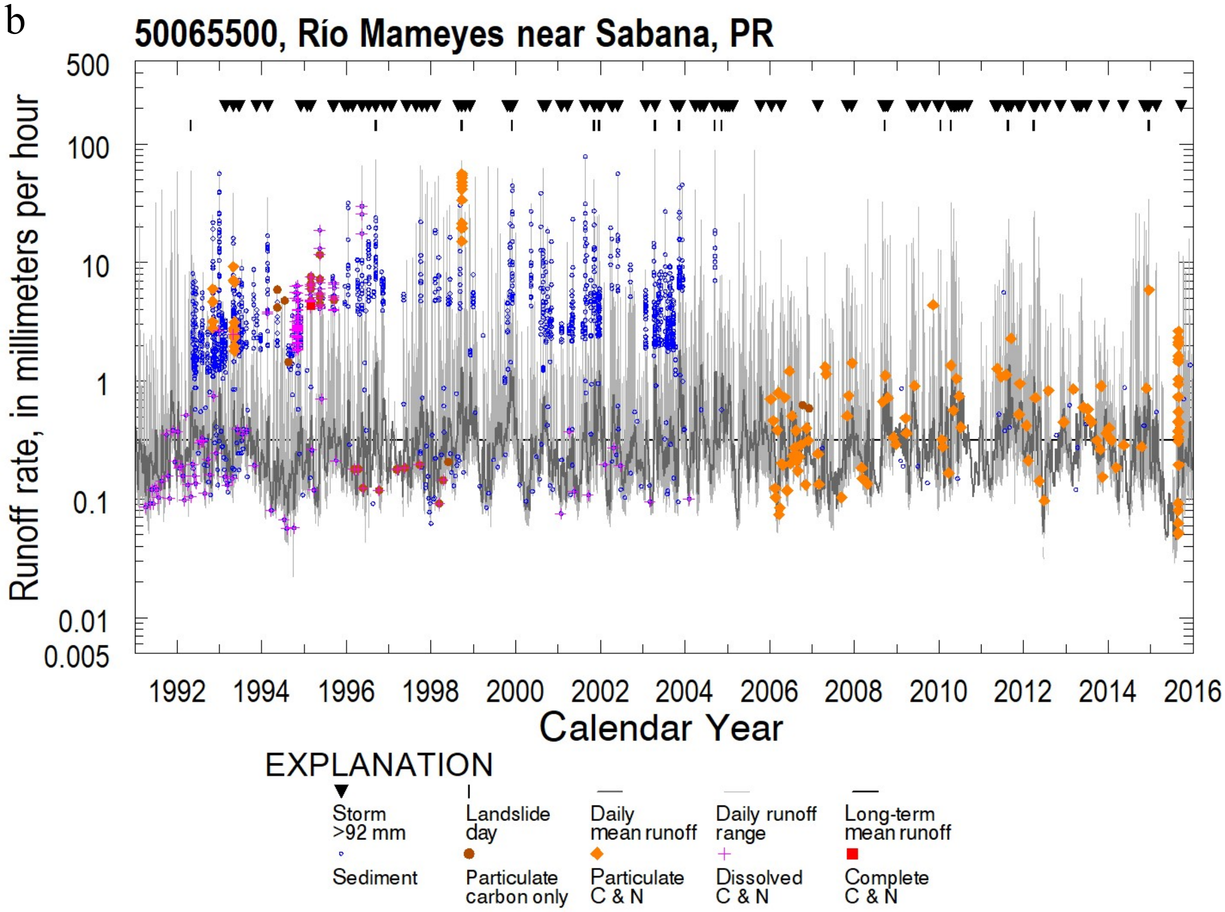

Supplementary Figure 2: River samples collected over the hydrograph, including extreme rainfall events, from 1991 to 2015. The study rivers were the a) Icacos and b) Mameyes, in the Luquillo Mountains, Puerto Rico. All sample with solid-phase analysis types (bottom row) include suspended solids. The inverted triangles represent large storms (>92 mm/event, as defined by Scholl and Murphy[1]). The small vertical tic marks represent landslide days as defined by Stallard[2]. The period 1991 through 2015 was sampled by the US Geological Survey (USGS)[3]. For the period 2006 through 2015 was sampled by the Luquillo Long-Term Ecological Research (LTER) Program and Luquillo Critical-Zone Observatory (LCZO) Program.

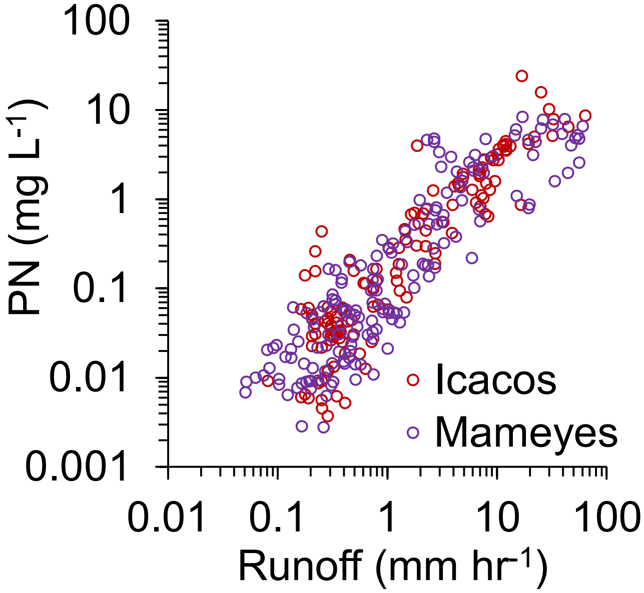

Supplementary Figure 3: River constituent relationship over a range of runoff for the study rivers. The relationship between measured river particulate nitrogen (PN, mg L^{-1}) relative to river runoff (mm hr^{-1}) for Icacos and Mameyes Rivers.

Tables

Supplementary Table 1: Sample and yield summary for Icacos and Mameyes Rivers from this study and previous reports

	Icacos	Mameyes
Area (km^2)	3.26	17.8
This study		
SS samples (n)	2158	1868
SS samples that did not meet section criteria (n)	32	58
SS samples used in LOADEST (n)	2126	1810
Measured POC samples (n)	194	199
Measured PN samples (n)	133	164
Estimated POC values (n)	1932	1611
Estimated PN values (n)	1993	1646
Mean runoff of SS samples used in LOADEST (mm hr^{-1})	5.7	4.8
Mean SS (mg L^{-1})	1038	274
Mean runoff of measured POC samples (mm hr^{-1})	5.6	5.3
Mean biogenic POC (%)	5.26	7.98
Mean biogenic POC (mg L^{-1})	27.8	14.5
Mean PN (%)	0.34	0.63
Mean PN (mg L^{-1})	1.35	1.02
Study by McDowell and Asbury[4]		
SS samples analyzed (n = x) [4]	127	-
Samples analyzed for POC (n = x) [4]	62	-
Samples analyzed for PN (n = x) [4]	54	-
Mean runoff at SS sample (mm hr^{-1}) [4]	0.45	-
Mean SS (mg L^{-1}) [4]	11.8	-
Mean POC[a] (%) [4]	3	
Mean POC[a] (mg L^{-1}) [4]	0.35	-
Mean PON (%) [4]	0.21	
Mean PON (mg L^{-1}) [4]	0.025	-
Max PON (mg L^{-1}) [4]	0.17	
1983-1985 runoff (mm yr^{-1}) [4]	3683	-
1983-1985 SS yield (t km^{-2} yr^{-1}) [4]	320	-
1983-1985 POC[a] yield (tC km^{-2} yr^{-1}) [4]	3.8	-
1983-1985 PN yield (tC km^{-2} yr^{-1}) [4]	0.2	-
Study by Murphy and Stallard[5]		
SS[b] samples used in LOADEST (n = x)) [6]	1408	1248

SS[b] (mg L^{-1}) [6]	560	118
Estimated biogenic POC (%) of SS [2,7]	1	1.9
1991-2015 runoff (mm yr^{-1}) [6]	3760	2750
1991-2005 SS yield (t km^{-2} yr^{-1}) [6]	2144	325
1991-2005 biogenic POC yield (tC km^{-2} yr^{-1}) [2]	22	4

[a] POC = Biogenic POC

[b] SS = SSol [6]

Supplementary Table 2: Regressions for estimating river biogenic particulate organic carbon (POC) or particulate nitrogen (PN) concentration (mg L^{-1}) in relation to suspended sediment (SS) concentration (mg L^{-1}) for Icacos and Mameyes Rivers, generated from measured POC and PN samples in Supplementary Data 1.

Site	Constituent	Equation	n	R^2	P*
Icacos	Biogenic POC	$POC_{est} = 0.054 \times SS^{0.977}$	194	0.96	< 0.0001
Mameyes	Biogenic POC	$POC_{est} = 0.069 \times SS^{0.977}$	199	0.91	< 0.0001
Icacos	PN	$PN_{est} = 0.004 \times SS^{0.944}$	133	0.96	< 0.0001
Mameyes	PN	$PN_{est} = 0.007 \times SS^{0.908}$	164	0.96	< 0.0001

*Correlation highly significant p < 0.001

Supplementary References

1 Scholl, M. A. & Murphy, S. F. Precipitation isotopes link regional climate patterns to water supply in a tropical mountain forest, eastern Puerto Rico. *Water Resour Res* **50**, 4305-4322 (2014).

2 Stallard, R. F. Weathering, landscape equilibrium, and carbon in four watersheds in eastern Puerto Rico, Ch. H in *Water quality and landscape processes of four watersheds in eastern Puerto Rico: U.S. Geological Survey Professional Paper 1789* (eds Murphy, S. F. & Stallard, R. F.) 199-248 (US Geological Survey, Reston, VA, 2012).

3 Murphy, S. F. & Stallard, R. F. Methods used to analyse water quality of four watersheds in Eastern Puerto Rico, Appendix 2 in *Water quality and landscape processes of four watersheds in*

eastern Puerto Rico: U.S. Geological Survey Professional Paper 1789 (eds Murphy, S. F. & Stallard, R. F.) 289-292 (U. S. Geological Survey, Reston, VA, 2012).

4 McDowell, W. H. & Asbury, C. E. Export of carbon, nitrogen, and major ions from three tropical montane watersheds. *Limnol Oceanogr* **39**, 111-125 (1994).

5 Murphy, S. F. & Stallard, R. F. Water quality and landscape processes of four watersheds in Eastern Puerto Rico. 292 (U.S. Geological Survey, Reston, Virginia, 2012).

6 Stallard, R. F. & Murphy, S. F. Water quality and mass transport in four watersheds in eastern Puerto Rico, Ch. E in *Water quality and landscape processes of four watersheds in eastern Puerto Rico: U.S. Geological Survey Professional Paper 1789* (eds Murphy, S. F. & Stallard, R. F.) 113-152 (US Geological Survey, Reston, VA, 2012).

7 Stallard, R. F. Data processing and computation to characterise hydrology and compare water quality of four watersheds in Eastern Puerto Rico, Appendix 1 in *Water quality and landscape processes of four watersheds in eastern Puerto Rico: U.S. Geological Survey Professional Paper 1789* (eds Murphy, S. F. & Stallard, R. F.) 263-287 (U. S. Geological Survey, Reston, VA, 2012).
