# Supplementary material for: Extreme rainstorms drive exceptional organic carbon export from forested humid-tropical rivers in Puerto Rico
Source: Nat Commun. 2022 Apr 19;13:2058. doi: 10.1038/s41467-022-29618-5 (PMC9018737; doi:10.1038/s41467-022-29618-5)
Supplement: Supplementary file 3 — Description of Additional Supplementary Files [file 41467_2022_29618_MOESM3_ESM.pdf]

Description of Additional Supplementary Files

File Name: Supplementary Data 1
Description: Measured river samples from Icacos and Mameyes, displayed in Figure 1, and Supplementary Figures

File Name: Supplementary Data 2
Description: Annual river yields determined from LOADEST for Icacos and Mameyes Rivers

File Name: Supplementary Data 3
Description: Global river suspended load yields, displayed in Figure 2a

File Name: Supplementary Data 4
Description: Interannual global petrogenic-carbon-poor bedrock river yields and NPP_{export} (%), displayed in Figure 2

File Name: Supplementary Data 5
Description: Summary of results and rainfall driven exports for Icacos and Mameyes Rivers, displayed in Figure 3

File Name: Supplementary Data 6
Description: Global river NPP_{export} (%), displayed in Figure 2b
